# Analysis and visualization of chromosomal abnormalities in SNP data with SNPscan

**DOI:** 10.1186/1471-2105-7-25

**Published:** 2006-01-18

**Authors:** Jason C Ting, Ying Ye, George H Thomas, Ingo Ruczinski, Jonathan Pevsner

**Affiliations:** 1 Department of Neurology, Kennedy Krieger Institute, Baltimore, Maryland 21205, USA; 2 Pathobiology Graduate Program, Johns Hopkins School of Medicine, Baltimore, Maryland 21205, USA; 3 Department of Pediatrics, Johns Hopkins School of Medicine, Baltimore, Maryland 21205, USA; 4 Department of Pathology, Johns Hopkins School of Medicine, Baltimore, Maryland 21205, USA; 5 Department of Genetics, Kennedy Krieger Institute, Baltimore, Maryland 21205, USA; 6 Department of Biostatistics, Johns Hopkins Bloomberg School of Public Health, Baltimore, Maryland 21205, USA; 7 Department of Neuroscience, Johns Hopkins School of Medicine, Baltimore, Maryland 21205, USA

## Abstract

**Background:**

A variety of diseases are caused by chromosomal abnormalities such as aneuploidies (having an abnormal number of chromosomes), microdeletions, microduplications, and uniparental disomy. High density single nucleotide polymorphism (SNP) microarrays provide information on chromosomal copy number changes, as well as genotype (heterozygosity and homozygosity). SNP array studies generate multiple types of data for each SNP site, some with more than 100,000 SNPs represented on each array. The identification of different classes of anomalies within SNP data has been challenging.

**Results:**

We have developed SNPscan, a web-accessible tool to analyze and visualize high density SNP data. It enables researchers (1) to visually and quantitatively assess the quality of user-generated SNP data relative to a benchmark data set derived from a control population, (2) to display SNP intensity and allelic call data in order to detect chromosomal copy number anomalies (duplications and deletions), (3) to display uniparental isodisomy based on loss of heterozygosity (LOH) across genomic regions, (4) to compare paired samples (e.g. tumor and normal), and (5) to generate a file type for viewing SNP data in the University of California, Santa Cruz (UCSC) Human Genome Browser. SNPscan accepts data exported from Affymetrix Copy Number Analysis Tool as its input. We validated SNPscan using data generated from patients with known deletions, duplications, and uniparental disomy. We also inspected previously generated SNP data from 90 apparently normal individuals from the Centre d'Étude du Polymorphisme Humain (CEPH) collection, and identified three cases of uniparental isodisomy, four females having an apparently mosaic X chromosome, two mislabelled SNP data sets, and one microdeletion on chromosome 2 with mosaicism from an apparently normal female. These previously unrecognized abnormalities were all detected using SNPscan. The microdeletion was independently confirmed by fluorescence in situ hybridization, and a region of homozygosity in a UPD case was confirmed by sequencing of genomic DNA.

**Conclusion:**

SNPscan is useful to identify chromosomal abnormalities based on SNP intensity (such as chromosomal copy number changes) and heterozygosity data (including regions of LOH and some cases of UPD). The program and source code are available at the SNPscan website .

## Background

A single nucleotide polymorphism (SNP) is a variation in a DNA sequence that occurs in an appreciable portion of the population. High density SNP microarrays provide information on the copy number of individual SNPs, based on measurement of the intensity of hybridized genomic DNA fragments to the microarray. These microarrays also provide genotype information on the state of heterozygosity or homozygosity at each SNP position. SNP arrays facilitate studies of chromosomal copy number change, large-scale linkage analysis, and whole genome association studies.

A variety of platforms for SNP-based genotyping are commercially available [1]. Most rely upon amplification of genomic DNA using the polymerase chain reaction (PCR) to reduce the complexity of the genome. One commonly used SNP microarray from Affymetrix consist of probesets corresponding to 11,555 SNPs at unique genomic loci (the Mapping 10 K array), with an average heterozygosity of about 37% and a median physical distance between SNPs of 105 kb [2]. Another Affymetrix platform contains 116,204 SNPs (the Mapping 100 K Set, consisting of the 50 K Xba and the 50 K Hind arrays that each contain ~58,000 SNPs) assigned to known loci. The Affymetrix GeneChip DNA Analysis Software 2.0 (GDAS) algorithm implements a dynamic model-based algorithm to genotype each SNP [2]. Each SNP genotype is assessed using 40 oligonucleotide probes organized into ten quartets consisting of probes representing both alleles. Four possible states or genotype models are assigned to each probe (A, B, AB, or NoCall), and a genotype call is assigned based on the confidence metric derived from the hybridization intensities to the probes [3-5]. Thus, this approach focuses on the conventional assumption that most SNPs are biallelic (having only two of the four nucleotides present at a given locus).

In addition to genotyping, a second category of information provided by SNP arrays is an estimation of the copy number of chromosomal DNA based on the hybridization intensity signals of genomic DNA samples. SNP arrays have been used to assess copy number changes such as aneuploidy, deletions, and cancer-associated amplifications [6,7]. The Affymetrix Copy Number Analysis Tool (CNAT) [2] displays SNP copy number values and corresponding probability (*p*) values across one chromosome, as well as genotype scores and *p *values as regions of LOH. Several recent reports describe the visualization of SNP array data using this tool or similar plots of intensity ratios and/or log_10 _*p *values (y-axis) versus chromosomal position (x-axis) [6-8]. These studies have validated the use of SNP arrays to measure chromosomal copy number changes.

A basic requirement of large-scale SNP assays is data visualization, which facilitates data analysis, statistical interpretation, and the identification of patterns of biological interest. We developed SNPscan as a tool for the visualization and analysis of high throughput SNP array data. Its main features are the ability to display the combined information of copy number and genotype, as well as associated *p *values, in a single plot for a dataset; the ability to simultaneously plot multiple data sets at user specified size; world wide web accessibility; paired (tumor vs. normal) ratio comparisons; and the ability to generate a file for visualization of SNP data on the genome browser developed by the University of California, Santa Cruz (UCSC) [9].

An additional feature of SNPscan is its ability to identify regions of uniparental disomy (UPD). UPD occurs when both copies of a chromosome are inherited from one parent [10]. Two identical copies of the same chromosome may be inherited from one parent (uniparental isodisomy), or two distinct copies of a particular chromosome may be inherited from the same parent (uniparental heterodisomy). UPD may occur across an entire chromosome or segmentally, following somatic events such as mitotic recombination. SNPscan is useful to identify uniparental isodisomy on the basis of a region of homozygosity with a normal copy number. Uniparental isodisomy can be discerned through analysis of an individual genomic DNA sample. Given the data from parental genotypes, uniparental heterodisomy can also be identified.

## Results

### Implementation of SNPscan

SNPscan was written in the Perl programming language [11], permitting uploads of the user's data, setting of the user's selections, and execution of functions implemented in the R programming language [12]. The R codes execute SNPscan's algorithm and generate graphics in several formats, to be sent back to the user via the SNPscan website [13] written in HTML and running under an Apache Web server.

#### Data input

The input data for SNPscan is a text file generated by Affymetrix CNATv2.1 software, via its export tool (with the first line containing the header "Copy Number Analysis Tool" deleted; this is necessary to allow R to interpret the data matrix). The platform may be the Affymetrix 10 K, 50 K Hind, 50 K Xba, or 100 K (both Hind and Xba) Mapping Arrays. (While SNPscan was designed for use with data from Affymetrix SNP arrays, SNPscan can be used with data from any platform, given the appropriate input format.) The text file has rows corresponding to SNPs. For 10 K arrays, the column headers are (1) row numbers, (2) Probe Set (i.e. SNP identifiers), (3) Chromosome, (4) Physical position, (5) ID_Call (e.g. for the identifier TC02_3387, the column header is TC02_3387_Call), (6) ID_SPA_CN (single point analysis of the copy number estimate), (7) ID_SPA_pVal (single point analysis of the significance of the copy number variation), (8) ID_CPA_pVal (continuous point analysis of the significance value; a significance value for the regional average), (9) ID_LOH (-log10 of the probability of continuous genomic region of homozygous calls). For each additional case analyzed by CNAT, columns (5) through (9) are repeated with values for that dataset. The SNPscan algorithm accepts a text file containing all this information, but does not analyze the ID_CPA_pVal data (note that ID_SPA_pVal information is used by SNPscan). For 50 K or 100 K arrays, columns (1) through (7) are as described above, followed by (8) ID_GSA_CN, (9) ID_GSA_pVal, and (10) ID_LOH. The SNPscan algorithm uses all these column values.

The columns that are used by SNPscan thus consist of SNP identifiers, chromosomal assignment and physical map position, and five additional columns for each array sample, as follows. (1) The call has four states: AA, BB, AB, or NoCall (i.e. no call, or null). These calls are determined in GDAS software using a dynamic model mapping algorithm, based on SNP intensity data [4,14]. (2) The copy number has values typically ranging from ~1 to 3, with a typical mean value of ~2.1 corresponding to disomic, autosomal loci and a mean of ~1.2 for hemizygous male X chromosome SNPs. (3) The negative log_10 _*p *value for copy number changes is a measure of the statistical significance of copy number changes. For example, a value of 4 corresponds to *p *< 10^-4 ^for rejecting the null hypothesis that no copy number changes have occurred at a given locus. The *p *values in GDAS are calculated by comparison of experimentally derived copy number values to values from 110 normal individuals. (4) An estimate of the statistical significance of copy number changes, accounting for the local regional changes in copy number. (5) A confidence metric of the genotype call, selected in GDAS by choosing the smallest *p *value from a Wilcoxon signed rank test to evaluate the four possible call models.

**Figure 1:**
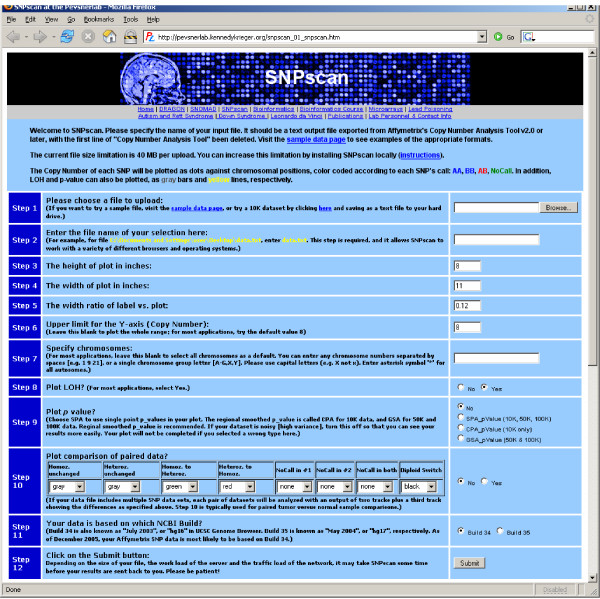
**Screen capture of SNPscan main page**. The web-based SNPscan program requires that the user upload a text file containing column headers with SNP identifiers and chromosomal position as well as SNP genotypes (AA, BB, AB, or NoCall), SNP copy number, and associated *p *values. This text file is obtained as an output from the Affymetrix CNAT. The SNPscan website includes separate upload pages for additional SNP analysis tools.

A typical SNPscan analysis, consisting of data on ~58,000 SNPs on ten individuals, is approximately 18 megabyte in size for file upload. We have chosen a default restriction of 40 megabytes to the file size for upload. (The availability of source code at the SNPscan website enables users to install the program locally and increase the file upload size.) There are three upload web pages, one for each of the SNPscan tools (described below); a screenshot of one upload page is shown in Fig. 1. Sample data files are provided at the SNPscan website to help users become accustomed to the tools [13]. SNPscan currently supports analyses using NCBI Builds 34 and 35.

#### Data output: SNPscanPlot

**Figure 2:**
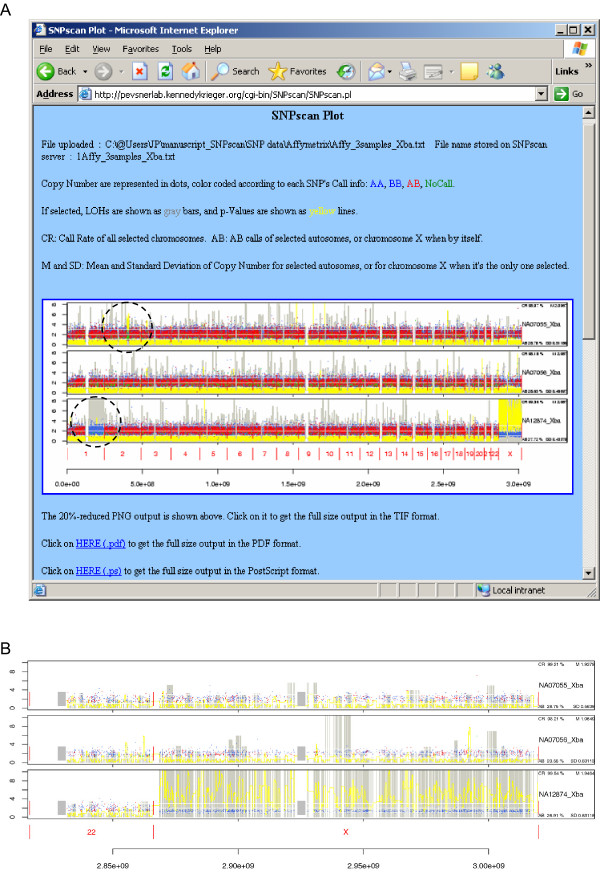
**SNPscanPlot visualizes data on SNP genotype and copy number**. A. Screen capture of SNPscanPlot output for three cases (NA07055, NA07056, NA12874). Each SNP array data set is organized in a row; options for types of input are 10 K, 50 K (Hind or Xba), or 100 K (combined Hind and Xba) arrays. The x-axis spans chromosomes 1–22 and X, with centromeres indicated (grey rectangles; see panel B for enlarged view). Note that a male case (bottom row) is easily distinguished from female cases by the profile of the hemizygous X chromosome, including many homozygous calls (blue dots), significant LOH -log_10 _*p *values (grey background), and a significant -log_10 _*p *value for copy number changes (yellow background). Also of note are a deletion on chromosome 2 (top row, dashed circle) and a region of UPD across chromosome 1 (bottom row, dashed circle). B. SNPscanPlot allows an expanded view of any chromosome(s); 22 and X are shown for the cases in panel A, highlighting the differences observed between male and female X chromosome SNP profiles.

**Figure 3:**
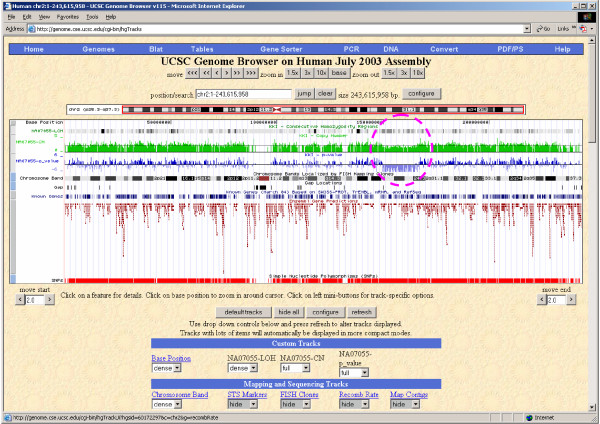
**Screen capture of SNP data visualized in the UCSC Human Genome Browser**. SNPscan converts a text file that has been generated by CNAT into a WIG file, which can then be visualized on the UCSC Genome Browser. For case NA07055 (shown in Fig. 2), 50 K Xba SNP data from chromosome 2 are displayed. The three custom tracks, arranged in rows, are LOH *p *value (color coded with more significant *p *values having a darker color), copy number (in green), and copy number *p *value (in blue). Note a region of deletion (dashed circle). The genome browser can be viewed for each chromosome at full scale or reduced to any region of interest. Additional tracks that are displayed in this example are chromosome band, gaps, known genes, Ensembl gene predictions, and SNPs. Dozens of additional tracks can be added or removed. Each WIG file that is generated can contain multiple SNP array data sets.

**Figure 4:**
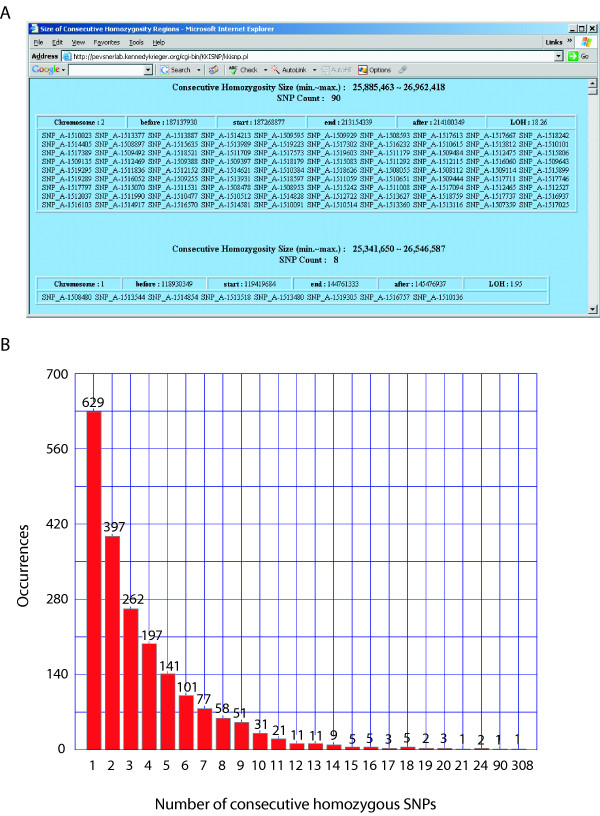
**SNPscan plots and tables for data analysis**. The SNPscan website offers a set of tools for the analysis genotype calls, including a description of SNPs in regions of consecutive homozygous calls (i.e. LOH) in table form (A) or with a plot (B). Both panels are screen shots; the labels in B have been redrawn for clarity.

There are three main outputs of SNPscan: a web page called SNPscanPlot (Fig. 2), a wiggle track (WIG) file that is used to visualize SNP data in a human genome browser (Fig. 3), and a series of plots and tables that summarize SNP genotype and copy number data (Fig. 4). For each SNP array dataset analyzed with the SNPscanPlot, the output includes the following features (Fig. 2A,B). The panels arranged vertically correspond to individual samples (e.g. subjects). Within each individual, the y-axis of the plot has three meanings, displayed with dots, bars, and connected lines of varying colors. The first meaning corresponds to copy number, represented by dots, with a user-defined scale (the default y-axis range for copy number values is 0–8). The second meaning is the grey vertical bar corresponding to LOH (confidence metric of the genotype call). The third meaning of the y-axis is the absolute value of the log_10 _*p *value of the single-point copy number (connecting yellow line). The points, in addition to representing the copy number, are also color-coded according to the possible calls: red (heterozygous call, AB), blue (homozygous call, AA or BB), and green (NoCall).

This combination of allelic calls with copy number is a unique feature of SNPscan. The sequence of plotting (LOH, *p *value, homozygous calls, heterozygous calls, NoCalls) is also uniquely arranged. Combining these two designs, SNPscan can be used to display and thus discover a variety of chromosomal anomalies. As an example, apparent homozygosity in the SNP output (e.g. an AA or BB call) can be due to true homozygosity, or to a hemizygous deletion (e.g. an A or B call) that is interpreted as homozygous by the GDAS software. Interpretation of SNPscan plot outputs can resolve this issue for stretches of homozygosity by simultaneously displaying the copy number (and its associated *p *value) along with the genotype. For authentic homozygosity, the copy number is not changed, while for hemizygous deletions, the copy number is reduced and there is a significant -log10 *p *value.

Fig. 2B shows a typical higher-resolution graphical output for a female case (upper two panels) and a male case (bottom panel) on chromosomes 22 and X. The single (hemizygous) male X chromosome has a large grey region (highly significant LOH *p *values), many blue dots (AA and BB calls) few heterozygous (red) points, and an apparent copy number loss (typically in the range 0.8–1.8) relative to chromosome 22 and relative to the female X chromosomes. For each chromosome, a short bar indicates the position of the centromere.

**Figure 5:**
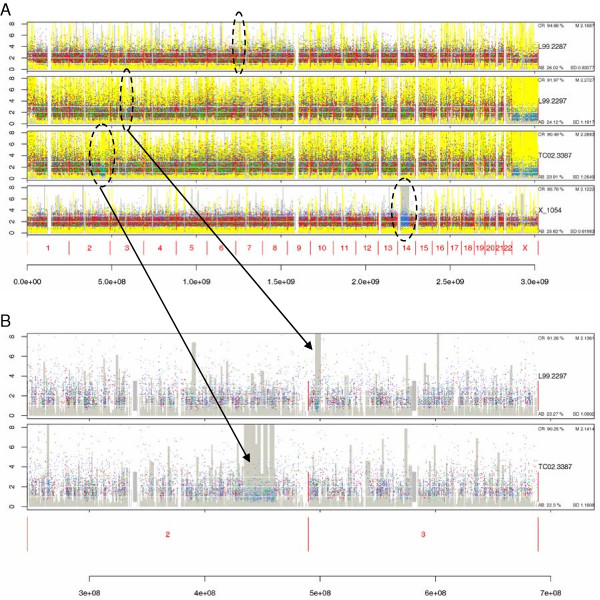
**Use of SNPscanPlot to visualize microdeletions and UPD**. A. SNPscanPlot visualization of cases involving microdeletions on chromosome 7 (top row; case L99-2287), 3 (second row; case L99-2297) and 2 (third row), and uniparental isodisomy of chromosome 14 (fourth row; case X_1054). The x-axis spans chromosomes 1–22 and X. B. Detailed view of microdeletions on chromosome 3 (top row) and 2 (bottom row). The x-axis spans chromosomes 2 and 3.

Each data set (track) in SNPscanPlot includes four statistics in the labeling area at the right of the plot. These are (clockwise from the upper-left corner, for whichever autosomes are selected): the call rate (i.e., 1 – NoCalls / all calls), the mean, the standard deviation, and heterozygosity rate (AB calls over (AA+AB+BB calls) (e.g. Fig. 2A,B and 5A,B). The call rate information includes all selected chromosomes. The mean, the standard deviation, and the heterozygosity rate (labelled as "AB") include only selected autosomes, unless chromosome X is the only selected chromosome. These values can be used to assess the chromosome copy number in a given chromosome (discussed below).

#### Graphical output file formats

Each SNPscanPlot result can be provided in several formats. The server returns a portable network graphics (PNG) output at a 20% reduction in size, to better fit into the screen, for a quick overview (Fig. 2A). The user can click on the PNG figure to obtain a tagged image file format (TIFF) file displayed at the full size as specified by the user (Fig. 1). This allows a more detailed view, but is slower for download due to the larger file size. Links are also provided to obtain a portable document format (PDF) file, as well as a PostScript (PS) file, to permit extremely detailed viewing and printing (e.g. Fig. 2B). The file size for a 50 K SNP array data set for ten individual arrays is typically 800 kilobytes for PNG, 2.7 megabytes for TIFF, 40 megabytes for PDF, and 55 megabytes for PS.

#### Data output: genome browser

The SNPscan program allows input data files to be converted to the WIG file format. This allows display of continuous-valued data in a track format that is compatible with the UCSC Genome Browser [15]. An example is shown in Fig. 3. Four custom tracks, visualized by uploading the WIG file to the UCSC Genome Browser website [9], are shown for chromosome 2. Many dozens of additional tracks may be added to the browser, and the data may be explored from a scale of several bases to the entire length of a chromosome.

#### Data output: plots and tables

A third feature of SNPscan is a group of graphical and tabular summary statistics to describe the genotype and copy number calls from a SNP data set. Examples include a tabular listing of SNPs organized by the size of a group of homozygous calls (Fig. 4A), and a plot of the number of instances of blocks of homozygous SNPs as a function of the length of the group (Fig. 4B).

### Identifying microdeletions via SNPscan

We analyzed a series of cases to demonstrate and validate the use of SNPscan. These cases include microdeletions, UPD, and possible chromosomal mosaicisms (presence of two or more cell lines, or subsets of a cell line, showing different chromosome constitutions).

#### Case 1: deletion on chromosome 7

**Figure 6:**
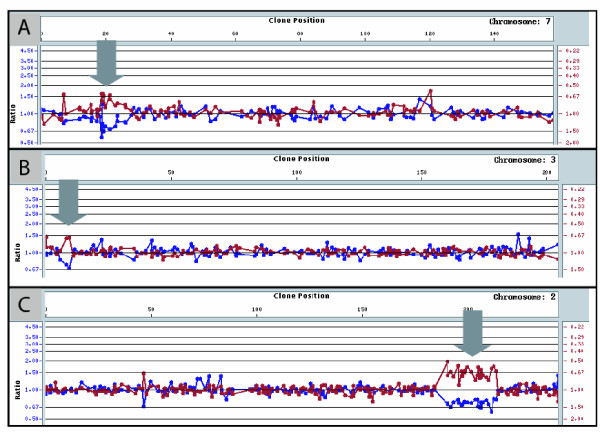
**Genomic microarrays reveal chromosomal microdeletions**. Genomic microarrays containing 2,600 BAC probes were used to assess microdeletions. The y-axis indicates ratio of patient to control signal and (in a dye swap paradigm) control to patient signal. The x-axis indicates physical map position (units in Mb). Panel A: case L99-2287 (chromosome 7 microdeletion in facial dysgenesis). Panel B: case L99-2297 (chromosome 3p deletion syndrome). Panel C: case L99-2287 (deletion of chromosome 2 including CPS I deficiency). Arrows indicate region of deletion. The plots were generated using SpectralWare (Spectral Genomics) software.

Hoover-Fong and colleagues described a female neonate with a unique phenotype of severe facial anomalies including anophthalmia, cryptophthalmos, bilateral cleft lip, and unilateral cleft palate [16]. Conventional karyotyping and fluorescence *in situ *hybridization (FISH) revealed a 7p15.1-21.1 deletion [16]. We isolated genomic DNA from a lymphoblastoid cell line (identifier L99-2287) and generated SNP data using both 10 K and 100 K mapping arrays. Using SNPscan, we visualized the 7p deletion region (Fig. 5A, top row). Note the presence of a reduced copy number, a preponderance of heterozygous calls (blue dots) with almost no heterozygous calls (red dots), and a high -log_10 _copy number *p *value (i.e. a significant *p *value). We estimated the size of the deletion by analyzing the CNAT-derived calculation of the CNAT copy number value, single point analysis copy number *p *value, and *p *value for LOH region based on genotype calls. Together, these parameters define the beginning and end positions of deleted regions. For this case, the size of the deletion was 6.7–9.2 megabases (Mb) based on 10 K, 50 K Xba and 50 K Hind SNP arrays (data not shown), further refining it from a reported estimate of 14 Mb [16]. In addition, we used genomic microarrays (array comparative genomic hybridization) in a dye-swap protocol to independently confirm the extent of the deletion on chromosome 7p (Fig. 6A). The genomic array result suggests an interstitial deletion of 7.6 Mb, consistent with the SNP data analysis.

The clinical anomalies observed in this case result from a developmental insult between four and seven weeks of gestation. A description of the exact genes that are deleted in this syndrome is necessary to elucidate the etiology of the disorder. Generation of a WIG file and display of the deletion region on the UCSC genome browser provided a view of the genomic landscape including the affected genes (data not shown).

#### Case 2: deletion on chromosome 3

Cargile et al. identified a case with 3p deletion syndrome, with the karyotype 46,XY,del(3)(p26.2).ish del(3)(p25.3p26.2)(3ptel+)[17]. Clinical features, typical for deletion 3p syndrome [18,19], included ptosis, microcephaly, growth retardation, and developmental delay. FISH studies narrowed the deleted region to 4.4 Mb, the smallest ever identified for this syndrome. We obtained genomic DNA from a lymphblastoid cell line (L99-2297), generated 10 K and 100 K SNP array data, and identified the 3p deletion region. A region of blue (homozygous calls) and green (NoCall) dots, with a copy number below 1, is evident in the whole-genome view (Fig. 5A, second row) and at higher magnification (Fig. 5B, top). LOH of SNPs indicated a deleted region of 2.9 to 4.5 Mb. Analysis of genomic microarrays suggested an interstitial loss of 3p from RP11-21J23 to RP11-91K4 (1.3 Mb), consistent with SNP data (Fig. 6B). This patient lacks many major clinical features that are typically present in 3p deletion syndrome, such as cardiovascular defects, renal anomalies, triangular face and rocker bottom feet. Thus it is of interest to define the genes that are deleted in this case, as well as the ones that are spared.

#### Case 3: deletion on chromosome 2

An interstitial deletion of chromosome 2q32-34 associated with carbamoyl phosphate synthetase I (CPS I) deficiency, a urea cycle defect, was reported by Loscalzo and colleagues [20]. We obtained a skin fibroblast cell line (TC02-3387), purified genomic DNA, and performed 50 K Xba SNP mapping arrays and array comparative genomic hybridization (aCGH; genomic microarrays). In this case a deletion region of approximately 32.2 Mb was refined to 26.1 Mb using SNP arrays (Fig. 5A, third row; Fig. 5B, lower panel), and to 23.3 Mb by genomic arrays (Fig. 6C). The region of deletion is characterized by a large grey area (indicating a significant LOH *p *value), by many blue dots (homozygous calls) and green dots (NoCalls). The few red dots (11 heterozygous calls out of 558 SNPs in this region) are consistent with the error rate on this individual's X chromosome (10 heterozygous calls out of 1,184 SNPs). The copy number of the deletion region is 1.41, comparable to the X chromosome copy number (1.35) and less than the nondeleted portion of chromosome 2 (mean value 2.22).

### Identifying uniparental isodisomy via SNPscan

Uniparental isodisomy can be detected in the analysis of SNP array data by the presence of regions of homozygosity (i.e. LOH) in the absence of copy number changes. We applied SNPscan to the analysis of a previously identified patient (case 4, below) and apparently normal individuals from the CEPH collection (cases 5 and 6). Altug-Teber et al. [21] recently used Affymetrix 10 K mapping arrays to analyze complete or segmental UPD in six families with diagnoses of Prader-Willi syndrome (matUPD15), Angelman syndrome (patUPD15), Silver-Russell syndrome (matUPD7), Beckwith-Wiedemann syndrome (patUPD11p), pseudohypoparathyroidism (patUPD20q) and a chromosomal rearrangement (patUPD2p, matUPD2q). We analyzed these data in SNPscan and observed uniparental isodisomy consistent with the findings of Altug-Teber et al. (data not shown).

#### Case 4: 14q uniparental isodisomy in a patient with a translocation

Antonarakis and colleagues described a nine-year old female with a de novo Robertsonian translocation t(13;14), short stature, developmental delay, and other symptoms [22]. Genotyping of polymorphic markers indicated maternal UPD for chromosome 14, including isodisomy for proximal markers and heterodisomy for distal markers. Additionally, there was mosaic trisomy 14 detected in 5% of blood lymphocytes. We obtained a lymphoblastoid cell line (identifier X_1054), purified genomic DNA and performed SNP array analysis. Uniparental isodisomy was evident by a large region of homozygous calls (blue dots), high LOH (grey background), and significant *p *values (yellow lines)(Fig. 5A, bottom row). We calculated that the copy number for chromosome 14 was 2.19 (3.2% higher than the copy number for other autosomes in this case); however, we did not independently confirm the presence of trisomy in these lymphoblasts, and in general this limited degree of mosaic trisomy does not permit visual identification of subtle copy number changes.

#### Case 5: uniparental isodisomy in an apparently normal female

**Figure 7:**
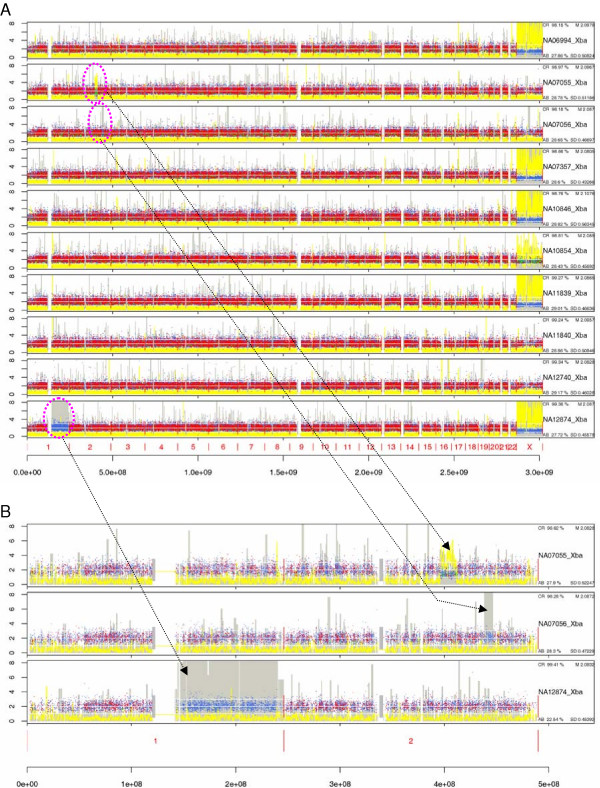
**Use of SNPscanPlot to visualize chromosomal anomalies in apparently normal individuals**. A. SNP array data for ten individuals, including some chromosomal abnormalities detected using SNPscan. These include: mosaic microdeletion on chr2q for NA07055 (second track from the top, dashed oval); UPD on chr2q of NA07056 (third track, dashed oval); mosaic chromosome X of female NA10854 (sixth track); UPD on chr19p on NA12740 (ninth track); and UPD on chr1q of NA12874 (tenth track, dashed oval). B. Enlarged view of chromosomes 1 and 2 for three individuals show in A; arrows indicate the affected regions.

We tested the functionality of SNPscan by visualizing SNP data from 90 individuals (30 trios of individuals and parents) assayed by Affymetrix on its 100 K SNP mapping set. The data are publicly available (downloaded on May 11, 2005)[23]. These samples are CEPH trios from the International HapMap Project [24] with no known chromosomal disorders. A group of ten CEPH SNP data sets visualized with SNPscan is shown in Fig. 7A. In case NA12874, a male with at least six grandchildren, we observed uniparental isodisomy across the entire long arm of chromosome 1 (Fig. 2A, bottom panel; Figs. 7A and 7B, bottom panels). For all apparently normal CEPH cases we studied for which we observed chromosomal abnormalities, there are no known instances of unreported kinship [24].

To confirm that case NA12874 had an extended region of homozygosity, we amplified genomic DNA from that case and five randomly selected CEPH controls. We generated and sequenced eight PCR products, four from chromosome 1p (12 SNPs that were from a presumably unaffected region of the chromosome) and four from 1q (11 SNPs that formed part of the presumed UPD region)(Table 1). The SNP calls were consistent with the sequenced DNA except for one SNP (SNP_A-1692270), in which all DNA sequences were homozygous while all SNP array calls were heterozygous. That discrepancy is likely explained because the 25 nucleotides spanning that SNP are expected to hybridize to two distinct positions on chromosome 1, based on BLAST searching.

For case NA12874, 11 of 11 SNPs on chromosome 1q were homozygous, while only 7 of 10 SNP positions on 1p were homozygous (Table 1). By comparison to five controls, this result is not likely to have occurred by chance (chi squared test, p < 0.01). For the SNP sequences on the 1p arm, the results for case NA12874 included three positions of heterozygosity, and did not differ from five control cases (p < 0.74). Thus, DNA sequencing of genomic DNA confirmed the homozygosity on 1q for N12874, consistent with an interpretation of uniparental isodisomy as visualized by SNPscan.

The copy numbers and call rates typically vary between experiments. For the ten apparently normal individuals in Fig. 7A, the mean autosomal copy number values ranged from 2.08 to 2.11 (standard deviation from 0.43 to 0.56), with call rates from 98.1% to 99.4%. For the cases in Fig. 5A, the mean autosomal copy number values ranged from 2.12 to 2.29 (standard deviation from 0.62 to 1.25), with call rates from 90.5% to 95.8%. SNPscan is useful for identification of abnormalities with datasets of varying call rates.

#### Case 6: Partial 2q uniparental isodisomy

Inspection of SNPscan visualization of the 30 CEPH trios revealed a possible case of partial uniparental isodisomy on chromosome 2q in case NA07056, a 65-year old female with at least six grandchildren (Fig. 2A,B middle panels). SNPscan showed a small segment in chromosome 2q of NA07056 as homozygous. Using the table and plot tool, we determined that region includes a stretch of 168 homozyogous SNPs in Hind 50 K array, and 170 SNPs in the Xba 50 K array. The length of this region was 7.98 Mb.

### Identifying mosaic chromosomal deletions via SNPscan

Mosaic chromosomal gains and losses complicate SNP analysis because somatic tissues may express variable chromosomal changes between cell types or within a population of cells. SNPscan can detect some cases of mosaicism as well as related problems such as mislabelled samples.

#### Case 7: 2q Deletion with mosaicism found in an apparently normal control

**Figure 8:**
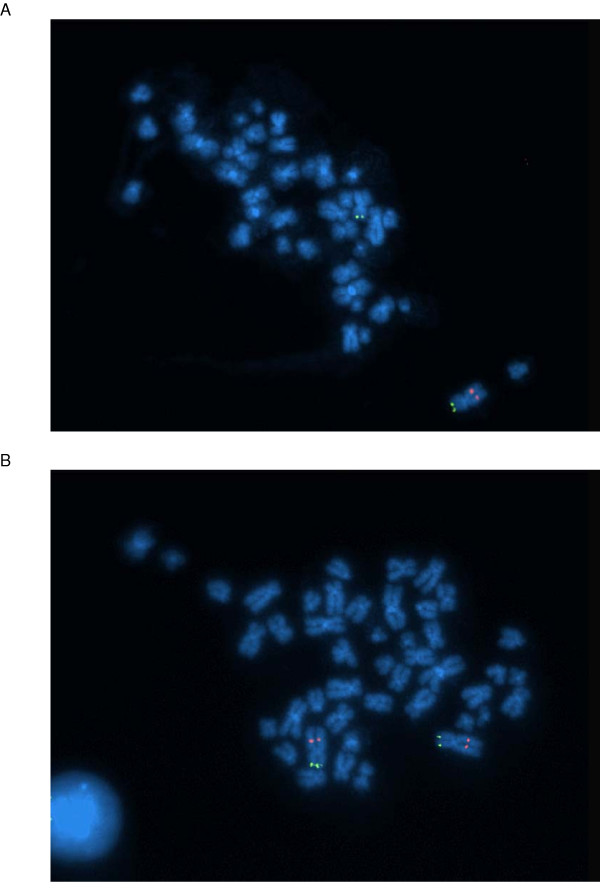
**FISH result showing mosaicism for a chromosome 2 microdeletion**. SNPscanPlot analysis suggested a microdeletion in chromosome 2q of case NA07055. 16 of 23 cells revealed a deletion (an example is shown in A), while the remainder appeared euploid (B).

By using SNPscan to analyze the 100K SNP data from apparently normal CEPH controls, we identified a previously unreported microdeletion on chromosome 2q24 (7.8 MB in size) of a healthy Caucasian female (Coriell repository identifier NA07055)(Fig. 2A, top panel; Fig. 7A, second panel; Fig. 7B, top panel). There was a region of homozygosity, with only three AB calls among a span of 158 consecutive SNPs from the Hind chip, and 21 AB calls among 189 consecutive SNP calls from the Xba chip. For this putative deletion region, the copy number was 1.66. (For all other autosomes, the mean copy number was 2.11.) There was an increased proportion of no calls in this region (15 no calls per 189 SNPs for the Xba array or 7.9% relative to 1.0% no calls across all autosomes), also consistent with the occurrence of a hemizygous deletion. We used FISH to confirm the presence of a deletion. Using two BAC probes (RP11-350H9 and RP11-1105K12), 16 of 23 metaphase cells (70%) displayed a deletion in 2q24, while the other 7 metaphase cells displayed both copies. An example of each case is shown in Fig. 8. These results suggest that the individual had a mosaic deletion in this region. This is consistent with the information displayed by the UCSC Genome Browser (Fig. 3), where the CN (copy number) track had lower values, the p_value track indicated the statistical significance of the copy number change, but the LOH track showed many fragmented bars of light color (instead of a large dark block shown in typical deletion segments) due to the mosaic nature of this cell line. In an independent study, researchers at the Coriell Cell Repositories performed aCGH using Spectral Genomics microarrays on this cell line (GM07055)(Dr. Patrick Bender, personal communication). A deletion of 8.7 Mb was observed, consistent with our findings.

#### Case 8: identifying loss of the X chromosome with mosaicism

**Figure 9:**
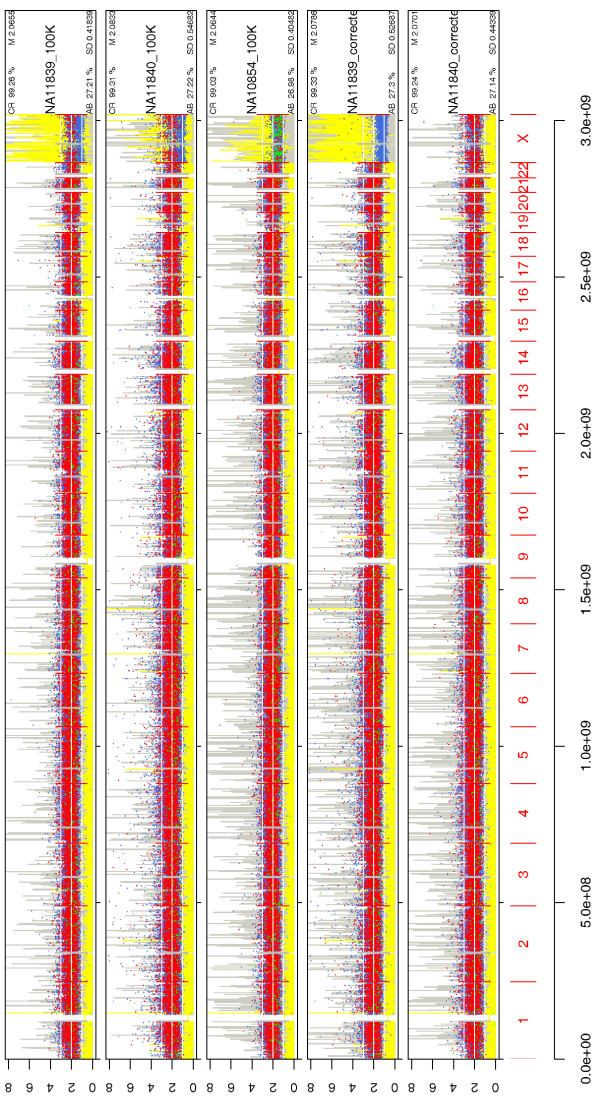
**SNPscan result for three apparently normal individuals using 100 K data**. SNPscan was used to analyze 100 K SNP data, combined from Affymetrix's 50 K Hind and 50 K Xba via CNAT v2.0. The first three tracks are a trio (father, NA11839; mother, NA11840; daughter, NA10854). Note that for both of the first two tracks, the patterns observed on the X chromosome are atypical. The first two tracks show a unique and apparently abnormal pattern of red dots in the copy number range that is normal for females (1–3), with blue dots exposed in a lowered copy number range that is normal for males (1–2). The *p *values (yellow area) for the first track are much higher than that of the second track, presumably because of the smaller number of heterozygous calls derived from the Hind array (see Table 3). Together, these observations suggest a pattern of SNPs that represents a combined male and female profile; our interpretation is that the labels were switched on two of the Hind datasets (see Table 3). The third track, showing data on an apparently normal female, has another pattern: many red and green dots mostly in the copy number range of 1 to 2.5, with *p *values (yellow) intermediate between normal female and male values. This represents a female with a mosaic loss of the X chromosome. After the labeling error was corrected for the first two tracks, the results (NA11839 as male and NA11840 as female) were plotted in the last two tracks.

Analysis of case NA10854 with SNPscan indicated a loss of one copy of the X chromosome, with mosaicism (Fig. 9). NA10854 was a 42-year old Caucasian female with 8 children. Of the 90 CEPH individuals, the mean copy numbers for all 44 male X chromosomes was 1.205 for Hind and 1.279 for Xba 50 K arrays, whereas the mean copy number for all 46 female X chromosomes was 2.018 for Hind and 2.093 for Xba. For case NA10854, the copy numbers were 1.429 for Hind and 1.552 for Xba. This finding would be expected if 70% of the cells from this individual had only one copy of chromosome X. Subsequent karyotyping by the Coriell Cell Repositories indicated that an X chromosome was missing from 68% of the cells, with a karyotype 45,X34/46,XX16 (Dr. Jay Leonard, personal communication).

Loss of a copy of the X chromosome can occur in females in vivo as a function of age, and in cell cultures in vitro as a function of population doublings. Further examination of SNPscan data revealed three other females (NA07348, NA10859, NA12145) with low chromosome X copy numbers, as listed in Table 2. These likely also represent females having mosaic loss of the X chromosome as revealed by SNPscan.

#### Case 9: identifying mislabelled SNP data

When displaying the 100 K trio data downloaded from Affymetrix's website, SNPscan revealed several anomalous results for NA11839 (father of NA10854) and NA11840 (mother of NA10854), as shown in Figure 9. For the NA11839 (male) Hind 50 K data, there were unexpectedly 263 heterozygous calls and a copy number of 2.031 for the X chromosome, typical of a female SNP profile. For the NA11840 (female) Hind data set there were zero AB calls and an X chromosome copy number of 1.222, typical of a male SNP profile. The Xba 50 K array data appeared appropriate for each gender. We concluded that the labels on the Hind SNP data sets were likely erroneous. After swapping them (Fig. 9, bottom two panels) the apparent error was corrected. The AB calls and averaged copy number of chromosome X, listed in Table 3, also confirmed these labelling errors.

### Paired data comparison via SNPscan

In some applications of SNP technology, paired samples (e.g. cancerous versus normal tissue) from one individual are analyzed. If the paired data option is selected, SNPscan generates a third plot for every two SNP data set inputs, in the input #1 vs. input #2 order. The third plot provides a series of comparisons between the samples.

#### Case 10: paired samples

**Figure 10:**
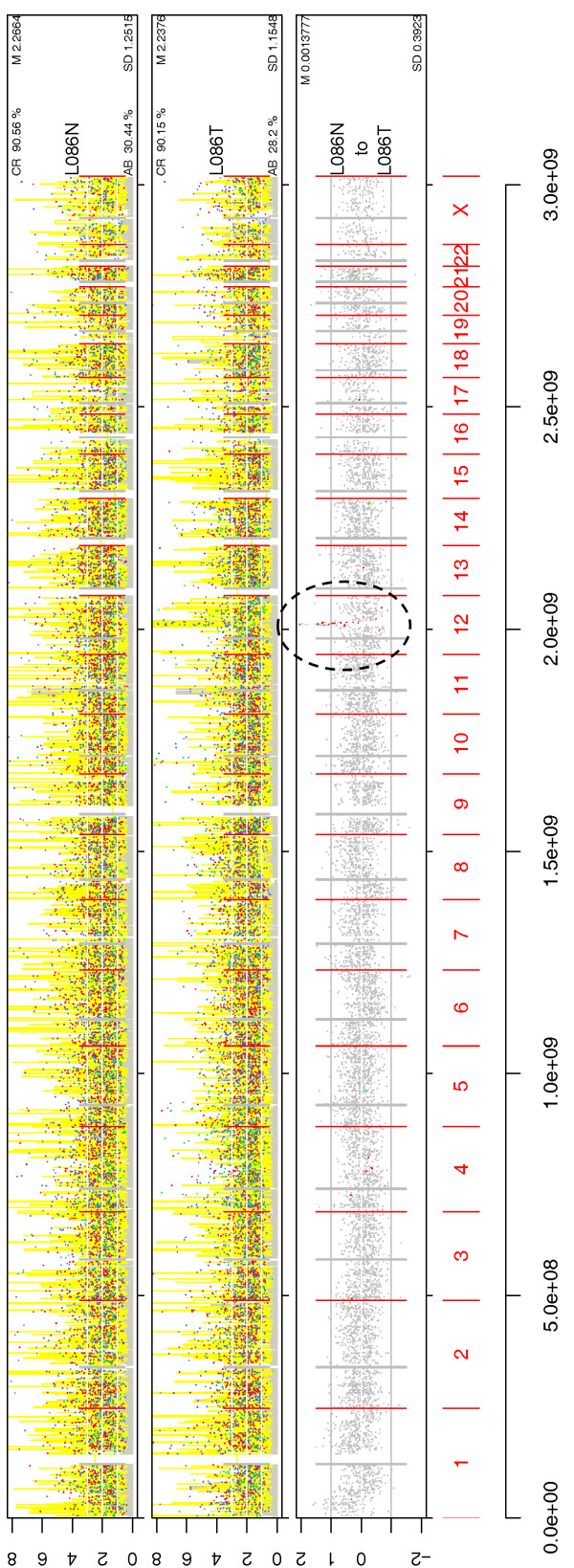
**SNPscanPlot for paired data**. When the paired data option is selected, SNPscan treats each two data sets as a pair, and produces a third track in the form of a ratio plot (in log_2 _scale) comparing the two samples. This feature is useful for any paired samples such as SNP profiles in normal versus cancer tissue. In this example the genotype changes from homozygous to heterozygous were indicated in red, while other optional color displays were suppressed. This highlighted a region of chromosomal amplification.

We analyzed data from paired lung cancer and unaffected tissue (whole blood) from the same individual (Fig. 10). When the paired data comparison option is selected in SNPscan, the user must provide an even number of SNP data sets in the input file. SNPscan allows its users to choose plotting colors for eight types of information that are displayed to visualize differences between a paired set of samples. The eight categories are (1) retention of homozygosity (e.g. an AA genotype matched to AA in the second sample, or BB→ BB); (2) retention of heterozygosity (e.g. AB→ AB); (3) genotype change from homozygous to heterozygous (AA→ AB, BB→ AB); (4) LOH genotype change (AB→ AA, AB→ BB); (5) NoCall in the first sample but not in the second; (6) NoCall in the second sample but not in the first; (7) NoCall in both; and (8) diploid switch (AA→ BB, BB→ AA)(this is not expected to represent a biological phenomenon). Fig. 10 (third panel, dotted oval) highlights a narrow region of copy number gain (genomic amplification) in a tumor sample. Genotype changes from heterozygous to homozygous are highlighted in red, showing a cluster of such changes in the amplification region. This might have occurred due to the expansion of one allele resulting in apparent homozygosity.

### Comparison to existing tools

SNPscan can be compared to other algorithms and programs that perform the analysis and visualization of SNP microarray data. Various tools have been created to analyze SNP data, including the discovery and analysis of SNPs as well as predictions of functional aspects of SNPs (reviewed in [25]). A variety of reports describe SNP copy number and genotype (LOH) data in adjacent plots [6-8,26,27], highlighting the usefulness of SNP arrays in generating both types of information. A relatively limited number of tools provide data visualization features partially overlapping those of SNPscan, as follows.

#### Affymetrix's Copy Number Analysis Tool v2.1

SNPscan analyzes SNP data that have been processed by CNATv2.1, providing a variety of features for further analysis and visualization. CNATv2.1 provides a visualization tool showing neighborhood smoothed copy number, an associated *p *value, and LOH information on three separate tracks, for one chromosome of one individual at a time [2]. It does not provide the capability to efficiently scan for chromosomal anomalies in datasets from a large number of individuals. In contrast, SNPscan allows data from multiple individuals to be analyzed simultaneously, limited only by the size of the upload buffer. (Users with dozens or hundreds of SNP data sets can set up a local copy of SNPscan and increase the upload capacity.) Relative to CNATv2.1, SNPscan offers unique features including visualization of regions of uniparental isodisomy, tabular and graphic summaries of genotype and copy number data, and conversion of SNP data to the WIG format. CNATv2.1 allows conversion of SNP files to an integrated genome browser format, but does not permit direct upload to the UCSC genome browser, and only one metric (LOH, copy number, and associated p values) may be exported at a time.

#### dChipSNP

dChipSNP permits concurrent analysis of LOH and copy number changes in paired samples [28,29]. The three main functions of dChipSNP [30] include statistical inference to identify LOH regions, copy number analysis, and linkage analysis. The Copy Number Analysis tool of dChipSNP offers a raw, or an inferred, copy number track using multiple normal samples for reference [26]. The algorithm includes a hidden Markov model to make inferences about LOH, with four states in the model comparing normal and tumor samples (non-informative, no call, loss, and retention). dChipSNP is a highly useful program, but requires paired samples for its LOH analyses, making it particularly appropriate for comparisons of tumor DNA and matched controls. While SNPscan is platform-independent, dChipSNP use is restricted to the Windows operating system.

#### Copy Number Analyzer for Affymetrix GeneChip Mapping 100 K arrays (CNAG)

Nannya et al. introduced an algorithm for analysis of paired samples using data from Affymetrix GeneChip Mapping 100 K arrays [31]. The CNAG algorithm provides an improved signal-to-noise ratio for the detection of copy number changes through the use of a hidden Markov model. Its features include corrections for the length and GC content of PCR products used for array hybridization, and optimized selection of the reference samples. The output includes a chromosome ideogram (x-axis) versus a series of tracks (y-axis) containing copy number ratios, copy number inference from a hidden Markov model, and heterozygous SNP calls. CNAG thus is distinguished from SNPscan which displays combined copy number and genotype information. CNAG is available by download as a set of algorithms written in C++ for Microsoft Windows, but a web-based implementation is currently unavailable.

## Discussion

A variety of technologies have been applied to the measurement of chromosomal abnormalities including conventional karyotyping (e.g. Giemsa staining of metaphase chromosomes), FISH, and conventional and array-based CGH [32,33]. SNP arrays represent a high-density, high-throughput technique with the capacity for measuring highly informative allelic information from complex genomic DNA samples [34]. For SNP array data analysis, a major requirement is accurate measurement of the genotype call and the copy number. Affymetrix GDAS software provides calls using a dynamic model algorithm. GDAS includes a calculation of the probability of a stretch of homozygosity occurring at random in the dataset, based on the probability of a homozygous call for each SNP relative to a reference set of 110 individuals [2]. The GDAS and CNAT software further provide estimates of the chromosome copy number. *p *values are generated by comparison of observed single-point and genome-smoothed estimates of the copy number to a reference set of 110 ethnically diverse individuals [8]. Genome-smoothed estimates account for the behavior of neighboring SNPs in analyzing copy number and associated *p *values.

In addition to generating statistical estimates of genotype and copy number values in samples assayed on SNP arrays, it is also essential to visualize the data. We created SNPscan as a web-accessible tool to both analyze and visualize data sets that have been initially processed in GDAS and CNAT.

SNPscan is useful for the following six reasons. (1) SNPscan provides a variety of useful summary statistics. These include the mean autosomal copy number and standard deviation as well as the call rate and percent heterozygosity for each SNP dataset. The SNPscan plots and tables also offer a variety of summary measures of LOH and copy number changes (e.g. Fig. 4). (2) It is extremely helpful to visualize data across multiple individuals (e.g. Fig. 7) to assess whether observed changes are specific to particular samples. In some applications, it is also helpful to analyze paired (e.g. normal and tumor) samples (Fig. 10). (3) A main feature of SNPscan plots is the integration of copy number and genotype data. This is crucial in interpreting the genetic mechanisms could account for observed SNP data, including deletions and duplications. Visualization using SNPscan indicated a series of chromosomal abnormalities. We validated several of these by FISH, genomic DNA sequencing, and aCGH. A number of these chromosomal anomalies, such as mosaic loss of a portion of chromosome 2 (Fig. 7), and mosaic loss of the X chromosome in four apparently normal females (Fig. 7 and Table 2), would have been difficult to detect without an appropriate visualization tool. While SNPscan is useful to identify a variety of chromosomal abnormalities, it is also important to consider possible genetic mechanisms such as *de novo *mutations, inherited mutations, inbreeding, etc. (4) In addition to being web-accessible, SNPscan offers both low-resolution (PNG, TIFF) and high-definition (PDF, PostScript) graphical output formats at user-defined sizes. (5) As a further feature of SNPscan, SNP datasets can be converted to the WIG format for visualization with the UCSC Genome Browser [15]. This provides tremendous flexibility and depth in further exploring the genomic landscape of potentially affected regions. (6) SNP arrays also represent a powerful tool for the detection of UPD and in particular uniparental isodisomy, which appears as a region of homozygosity without copy number change. SNPscan permitted detection of UPD in a previously characterized clinical case (Fig. 5) and in an apparently normal case (Fig. 7). There are three main disease consequences that may be caused by UPD, all of which could occur in a single patient: trisomy mosaicism, homozygosity of recessive autosomal mutations, and genomic imprinting [35-37]. While UPD has only been appreciated as a genetic phenomenon since 1980 [10], it is likely that the analysis of high-density SNP data will enable the discovery of many more cases.

## Conclusion

SNPscan enables its users to convert high throughput, alphanumeric SNP data into plots of various sizes and resolutions for rapid visual identification of chromosomal anomalies. SNPscan uniquely correlates each SNP's copy number information with its allelic call information. The correlated data plotted across physical positions, aided with LOH and *p*-value information, generates distinct patterns for easy visual identification of a variety of abnormalities. SNPscan is freely available to the research community.

## Methods

### Acquisition of cell lines and DNA samples from patients and controls

Genomic DNA and lymphoblastoid cell lines were obtained from apparently normal individuals (Coriell Cell Repositories, Camden, NJ)[38]. Identifiers for Coriell DNA samples begin with the letters NA, while cell lines begin with GM. Lymphoblastoid cell lines were obtained from patients with known chromosomal abnormalities from the Kennedy Krieger Institute. Samples were deidentified, and informed consent was obtained in all cases. The study was conducted with Institutional Review Board approval from the Johns Hopkins University.

### Acquisition of SNP data

Genomic DNA samples (250 nanograms) were analyzed on Affymetrix, Inc. (Santa Clara, CA) SNP arrays [2]. The quantity and integrity of all DNA samples was assessed by electrophoresing an aliquot on an agarose gel (1%) and staining with ethidium bromide. SNP array data were generated at the Center for Inherited Disease Research during training on SNP array protocols (Johns Hopkins) or in the laboratory of Dr. David Sidransky (Johns Hopkins University). SNP microarrays consist of either the 10 K set or the GeneChip^® ^Mapping 100 K Set, consisting of a 50 K *Xba*I chip and a 50 K *Hind*III chip. Initial data analysis was performed with GeneChip DNA Analysis Software (GDAS) software and the Chromosome Copy Number tool (Affymetrix, Inc.). Additional SNP data sets were acquired from publicly available sources.

Affymetrix 100 K set data were analyzed with GDAS software. Quality control from Affymetrix includes Modified Partitioning Around Medoids (MPAM) Call Rate (MCR), which is applied to 10 K chips [39]. MPAM is a model-based algorithm to determine which allele is present at a particular SNP site in each sample. Models of the relative allele signal (RAS) value were developed based on the performance of over 110 presumably normal control samples. "Call zone" scores are used to partition scores into the categories AA, BB, AB, or NoCall. For the 100 K set Affymetrix SNP chips, a Dynamic Model Mapping (DMM) algorithm is applied. Probe cells are paired to provide a perfect match (PM) and mismatch (MM) 25 mer oligonucleotide. In all, for each SNP there are ten quartets, of which the optimal seven are used. Each quartet consists of a PM for allele A, mismatch for allele A, PM for allele B, and mismatch for allele B. The various quartets correspond to offsets of the mismatched nucleotide from the 13^th ^(central) position of the 25 mer oligonucleotide. DMM is a likelihood model based algorithm that uses Wilcoxon's signed rank test to provide a genotype call for each SNP, as well as quality information for each call.

### Use of SNPscan website

A typical use of the web-accessible version of SNPscan is as follows. From the home page of SNPscan, three main tools are available: SNPscan (to generate SNPscan Plots), KKISNP (to obtain summary tables and graphs), and SNPscan Browser (to create a wiggle file for upload to the UCSC Genome Browser). To use SNPscan, one follows the 12 steps described in detail on the website. Briefly, these steps are as follows. (1) Select a file to upload. This can be from a local machine, or from sample text files provided on the SNPscan website. (2) Enter the file name. (3)(4)(5) Set the height, width and width ratio of the output plots; default settings are provided for letter size paper. (6) Set the upper limit for the y-axis; the default setting of 8 is appropriate for most users. If left blank, the y-axis scales to the highest data values. (7) Specify the chromosomes. As a default, when this field is left blank, all chromosomes are shown. One can select a specific chromosome (e.g. 4) or set of chromosomes (e.g. 4, 5, 11), or group number (e.g. G for chromosomes 21 and 22). (8) Plot LOH; this is selected as a default. (9) Plot *p *value; this can be adjusted for 10 K or 50 K and 100 K analyses, or can be turned off. (10) Plot a comparison of paired data (e.g. paired normal versus cancer samples from individuals). For this feature, a third track is generated for each pair, highlighting their differences. There are eight optional color selections, with 11 colors in each palette, for features such as allelic gains and losses. (11) Select the NCBI Build (34 or 35). (12) Click the submit button. Depending on the size of the dataset and the network traffic, it may take several minutes for data to be returned. The output appears in the TIFF format, with options to select PNG, PS, and PDF formats.

### Acquisition of aCGH data

Chromosomal microduplications and microdeletions were assessed using CGH arrays (Spectral Genomics, Inc., Houston, TX)[40]. The SpectralChip 2600™ array consists of 2,600 BAC clones corresponding to all 24 human chromosomes. BACs are spaced at an average 1 Mb interval. Genomic DNA from each patient (test sample) and from a pool of normal individuals (reference sample) was fragmented by sonication (Misonix, Inc., Farmingdale, NY). 1 μg of genomic DNA from each patient sample was labeled with Cy3-dCTP or Cy5-dCTP (Amersham Biosciences, Piscataway, NJ) and hybridized according to a Spectral Genomics protocol. To confirm adequate dye labeling efficiency, samples were electrophoresed on a 1% agarose gel. Repetitive DNA sequences were blocked by the addition of Cot-1 DNA. Hybridization was for 16 hours at 37°C followed by a series of washes (2X sodium chloride sodium citrate, 0.5% sodium dodecylsulfate, 22°C, 5 seconds; 2X SSC, 50% formamide, 50°C, 20 min; 2X SSC, Igepal 0.1%, 50°C, 20 min; 0.2X SSC, 50°C, 10 min, then rinsed twice in H_2_O). Slides were dried with a stream of nitrogen gas, then scanned with a GenePix 4000B (Axon Instruments, Sunnyvale, CA). Image analysis was performed using GenePix Pro software to generate a GenePix Results (.gpr) file. The fluorescence ratio was determined in order to identify regions of genomic DNA that deviate from a 1:1 ratio. The .gpr file was analyzed using SpectralWare software (Spectral Genomics).

### Fluorescence in situ hybridization

FISH was performed on metaphase chromosomes by the method of Pinkel and Gray [41]. BAC clones were obtained from Roswell Park Cancer Institute (Buffalo, NY). 1 μg of BAC DNA was labelled by nick translation with a kit (Vysis Inc., Downers Grove, IL) including incorporation of Spectrum Orange dUTP. The BAC DNA was separated from the reaction mixture, co-precipitated with highly repetitive DNA, denatured, pre-annealed, then hybridized in situ to metaphase cells.

Lymphoblastoid cells were obtained from the Kennedy Krieger Institute (Baltimore, MD) or the Coriell Cell Repository (Camden, NJ). Cells were grown in RPMI medium, treated with colcemid (a mitotic spindle inhibitor), and metaphase spreads were incubated with chromosome-specific telomere probes (Vysis, Inc.) and labelled BAC clones. Cells were counterstained with 4',6-diamidino-2-phenylindole (DAPI) to visualize nuclei. Fluorescent signals were visualized using a Zeiss AxioSkop equipped with epifluorescence, appropriate filter sets, and a computer-assisted FISH capture system and software for producing high resolution images.

### Genomic DNA sequencing

Genomic DNA samples from five apparently normal cases (NA07357, NA06985, NA07056, NA10855, NA12006) and one case suspected to have UPD (NA12874) were purchased from Coriell Cell Repositories. Genomic DNA (100 ng) was PCR amplified (Expand High Fidelity PCR System, Roche Applied Science, Indianapolis, IN) using standard conditions including 30 to 35 cycles using denaturation (94°C, 15 sec), annealing (60°C, 30 sec), and extension (72°C, 1 min). The eight sets of oligonucleotide primers were as follows (numbered 1p1 to 1p4 for chromosome 1p region, and 1q1 to 1q4 for chromosome 1q): 1p1, 5'-CTCTGTGCAAGGTGTGAGGA-3' and 5'-ATGGCCCAAGGTCACATAAA-3'; 1p2, 5'-TTGAAACACTTCACAAAAGATGTG-3' and 5'-GTGCTCCTGGGAGAACTCAG-3'; 1p3, 5'-CCCAGTGCCATTATTACACTCA-3' and 5'-ATAGGGGCTCTGCACCTTTC-3'; 1p4, 5'-TGTATTGTTGGATTTGGTTTGC-3' and 5'-AGTCCCAGATGGGTTCACTG-3'; 1q1, 5'-TGTCTTCCAAAACGCACTTG-3' and 5'-AGCCCATCACGTCATATTCC-3'; 1q2, 5'-GGGGGTATCAGAGGCAATTT-3' and 5'-AGTGAAGAGCTCCTGCCTTG-3'; 1q3, 5'-CATCCGTGAGAATGGAAACC-3' and 5'-ATGAGGTCCATGCAGGAAAA-3'; 1q4, 5'-CAGGCAGGCTTTGACTCTTC-3' and 5'-CCCTAGAAACAGCTCCCAAA-3'. PCR products were electrophoresed on a 1.5% agarose gel, purified (Gel Extraction Kit, Qiagen, Valencia, CA), and sequenced (Synthesis and Sequencing Facility, Johns Hopkins). Eight sets of primers were designed to span 2 or 3 SNPs (selected with a bias towards SNPs having heterozygous calls) in <500 base pair regions of chromosome 1p (where UPD is not expected) and 1q.

### SNPscan availability and requirements

Project name: SNPscan

Project home page: 

Operating system(s): Platform independent website

Programming languages: Perl, HTML, R v2.1

Other requirements: Data input is from CNATv2.0 or v2.1 (Affymetrix).

License: GNU GPL

Any restrictions to use by non-academics: none. The source code is downloadable from the website.

## List of abbreviations

CEPH, Centre d'Étude du Polymorphisme Humain; CGH, comparative genomic hybridization; CNAT, Copy Number Analysis Tool; DAPI, 4',6-diamidino-2-phenylindole; FISH, fluorescence in situ hybridization; GDAS, GeneChip DNA Analysis Software; LOH, loss of heterozygosity; Mb, megabase; PDF, portable document format; PNG, portable network graphics; PS, PostScript; SNP, single nucleotide polymorphism; TIFF, tagged image file format; UCSC, University of California, Santa Cruz; UPD, uniparental disomy; WIG, wiggle track.

## Authors' contributions

JT designed the SNPscan algorithm, developed the KKISNP tools, and implemented the SNPscan website. He identified novel chromosomal anomalies in the apparently normal 90 individuals discussed in this paper via SNPscan and performed data analysis in R. YY performed the FISH studies and sequencing of genomic DNA, and contributed to writing the manuscript. GHT made intellectual contributions to the design and interpretation of the experiments and contributed to the writing of the manuscript. IR contributed to the data analysis and software development. JP conceived of the study and contributed to data analysis, development of the SNPscan website, and writing the manuscript. All authors read and approved the final manuscript.

## Figures and Tables

**Table 1:** Identification of a homozygous region by DNA sequencing and SNP array detection. For six individuals (columns), data were obtained at 23 positions along chromosome 2 based on direct DNA sequencing of PCR products ("seq" columns) or based on SNP array genotype calls ("call" columns). A region of homozygosity in case NA12874 is indicated (bold).

Nucleotide	NA07357		NA06985		NA07056		NA10855		NA12006		NA12874	
SNP_ID	**seq**	**call**	**seq**	**call**	**seq**	**call**	**seq**	**call**	**seq**	**call**	**seq**	**call**

SNP_A-1744333	C	AA	C	AA	C	AA	C	AA	C	AA	C	AA
SNP_A-1692270	A	AB	A	AB	A	AB	A	AB	A	AB	A	AB
SNP_A-1716769	C	AA	C/T	AB	C/T	AB	C	AA	C/T	AB	C	AA
												
SNP_A-1735604	T	BB	G	AA	T	BB	G	AA	G	AA	G	AA
SNP_A-1698365	C	BB	C/T	AB	C	BB	C	BB	C/T	AB	T	AA
SNP_A-1735398	G	BB	A/G	AB	G	BB	A/G	AB	A/G	AB	G	BB
												
SNP_A-1748755	C	BB	C/T	AB	C	BB	C	BB	C	BB	C/T	AB
SNP_A-1680534	C	BB	C	BB	C	BB	C	BB	C	BB	C	BB
SNP_A-1739459	C	BB	C/T	AB	T	AA	T	AA	C/T	AB	C	BB
												
SNP_A-1695373	G	BB	G/T	AB	G/T	AB	G/T	AB	G	BB	G/T	AB
SNP_A-1694479	C	AA	C/T	AB	C/T	AB	C/T	AB	C	AA	C/T	AB
SNP_A-1719671	C	AA	C/T	AB	C/T	AB	C/T	AB	C	AA	C/T	AB
												
SNP_A-1734876	C/T	AB	C/T	AB	C/T	AB	T	BB	C	AA	**T**	**BB**
SNP_A-1751485	G/T	AB	G	AA	G	AA	G	AA	G	AA	**T**	**BB**
SNP_A-1717899	T/C	AB	T/C	AB	T/C	AB	C	AA	T	BB	**C**	**AA**
												
SNP_A-1758974	T	BB	T	BB	T	BB	T	BB	T	BB	**T**	**BB**
SNP_A-1723176	T/G	AB	T/G	AB	T	AA	T/G	AB	T	AA	**T**	**AA**
SNP_A-1681286	C/A	AB	C/A	AB	C	BB	C/A	AB	C	BB	**C**	**BB**
												
SNP_A-1713946	C	BB	C	BB	C/T	AB	C/T	AB	C	BB	**T**	**BB**
SNP_A-1697831	A	BB	A/T	AB	A/T	AB	A/T	AB	A	BB	**A**	**BB**
												
SNP_A-1677784	C	BB	C/T	AB	C	BB	C	BB	C	BB	**C**	**BB**
SNP_A-1725840	A	AA	A/G	AB	A	AA	A	AA	A	AA	**A**	**AA**
SNP_A-1647716	G	AA	G/T	AB	G	AA	G	AA	G	AA	**G**	**AA**

**Table 2:** Possible mosaic loss of the X chromosome in cell lines from four females. SNPscan analyses revealed low X chromosome copy number values in four female cases. 44 males and 46 females comprise a group of 30 apparently normal CEPH trios. Values represent mean copy number ± standard deviation.

Case	X chromosome		Autosomes	
	Hind	Xba	Hind	Xba

NA10854	1.43 ± 0.25	1.55 ± 0.34	2.04 ± 0.34	2.09 ± 0.46
NA07348	1.63 ± 0.32	1.73 ± 0.34	2.05 ± 0.40	2.09 ± 0.46
NA10859	1.78 ± 0.35	1.89 ± 0.47	2.05 ± 0.40	2.10 ± 0.55
NA12145	1.79 ± 0.31	1.84 ± 0.38	2.05 ± 0.36	2.09 ± 0.47
Males (n = 44)	1.20 ± 0.06	1.28 ± 0.06	2.06 ± 0.02	2.09 ± 0.02
Females (n = 46)	2.03 ± 0.13	2.09 ± 0.12	2.06 ± 0.02	2.09 ± 0.01

**Table 3:** Identification of mislabelled samples. Mislabelled samples NA11839 and NA11840 (father and mother of NA10854, respectively) were first identified visually via SNPscan based on anomalous X chromosome profiles (Fig. 9). Subsequent analysis of genotype calls and copy number values suggested that the NA11839 Hind and NA11840 Hind labels (as shown in the table) were reversed; upon switching labels (not shown here; see Fig. 9, bottom two tracks), male case NA11839 has zero AB calls on the X chromosome and an X chromosome copy number of 1.22 as expected for a male. After correcting the labels, female case NA11840 has 621 AB calls on the X chromosome, and an X chromosome copy number of 2.09 as expected for a female.

	AA	AB	BB	NoCall	X Chromosome copy number
NA11839_Hind	420	263	455	12	2.031
NA11839_Xba	573	0	606	5	1.223
NA11840_Hind	560	0	587	3	1.222
NA11840_Xba	389	358	430	7	2.153
